# *CaCBP2* Negatively Regulates Pepper Resistance to *Phytophthora capsici* Infection

**DOI:** 10.3390/plants15030381

**Published:** 2026-01-26

**Authors:** Juan Du, Zhancheng Jia, Fangyu Qi, Binqian Tang, Huipin Yang, Xinhao Zhang, Qinbiao Yin, Jun Wang, Cheng Xiong, Xuexiao Zou, Zhuo Zhang, Feng Liu

**Affiliations:** 1 Longping Agricultural College, Hunan University, Changsha 410128, China; dujuan888666@outlook.com (J.D.); zou_xuexiao@163.com (X.Z.); 2 Yuelushan Laboratory, Changsha 410128, China; jzc@stu.hunau.edu.cn (Z.J.); bqtang@hunau.edu.cn (B.T.); hpyangx@163.com (H.Y.); 17267298729@163.com (X.Z.); yin18229425944@163.com (Q.Y.); wj1923535553@163.com (J.W.); xiongchenghzau@163.com (C.X.); 3 College of Horticulture, Hunan Agricultural University, Changsha 410128, China; qifangyu@sjtu.edu.cn; 4 Institute of Plant Protection, Hunan Academy of Agricultural Sciences, Changsha 410125, China

**Keywords:** *CaCBP2*, RNA-binding protein, *Phytophthora capsici*, transcriptome, WGCNA, negative regulator

## Abstract

Research on the CBP gene family in plants is scarce, with only sporadic reports on its association with immune responses. No systematic study has explored how CBP family genes regulate pepper resistance against *Phytophthora capsici*. Here, we focused on pepper *CaCBP2*, an RNA-binding protein, whose expression was significantly induced by *P. capsici*. Functional validation via VIGS and heterologous overexpression confirmed *CaCBP2* as a negative regulator of pepper resistance to *P. capsici*. Based on physiological assays, transcriptome sequencing and WGCNA, we speculate it may mediate immune responses by regulating antioxidant systems, defense hormone metabolism, and disease resistance-related genes. Our findings fill the relevant research gap, enrich the role of RNA-binding proteins in plant anti-phytophthora defense, and provide a novel target for crop disease-resistant breeding.

## 1. Introduction

Pepper (*Capsicum annuum* L.) is a globally cultivated cash crop with substantial edible, medicinal, and ornamental values [1,2,3,4,5]. However, its production is severely constrained by *Phytophthora capsici* (*P. capsici*), an oomycete pathogen that causes devastating pepper blight [6,7,8,9,10]. This soil-borne disease induces plant wilting and death, ultimately leading to massive economic losses in the global pepper industry [11,12,13]. Deciphering the molecular mechanisms underlying pepper resistance to *P. capsici* and identifying key resistance-related genes represents the most pivotal strategy for disease control.

Plant defense against pathogens depends on the coordinated regulation of multiple immune signaling pathways [14,15,16]. Among these pathways, calcium (Ca^2+^) signaling functions as a core second messenger [17,18,19]. It serves as a critical link that connects pathogen perception at the cellular level to the activation of downstream defense responses [20,21,22]. Notably, Ca^2+^-mediated expression of disease resistance genes requires the orchestration of fundamental molecular processes (e.g., transcription and mRNA processing), and the nuclear cap-binding complex (CBC) is a key molecular machinery involved in these processes [23,24,25,26,27,28]. The CBC is a heterodimer composed of two subunits: CBP20 (20 kDa) and CBP80 (80 kDa) [29,30,31,32]. *CBP20*, which contains a conserved RNA-binding domain (RBD), is involved in key processes including mRNA processing, miRNA biogenesis, and stress responses [33].

Recent studies have established that CBP family genes (the subfamily encoding CBC components) function as critical nodes bridging Ca^2+^ signaling and gene expression regulation in plant immunity. For instance, in Arabidopsis thaliana, *CBP60b* serves as a key transcriptional activator of immune responses, while *CBP60g* is activated via the synergistic action of Ca^2+^-signaling components (e.g., *TOUCH3*, calmodulins 1/4/6, and calcium-dependent protein kinases) to modulate antifungal resistance [34,35,36,37,38]. Despite these advances, research on the CBP family in Solanaceous crops remains extremely limited: no systematic investigations have addressed the role of pepper CBP genes in *P. capsici* resistance, leaving critical knowledge gaps regarding their functional relevance to immune regulation in pepper.

The pepper *CaCBP2* (the focus of this study) is an RNA-binding domain (RBD)-containing protein; preliminary bioinformatic predictions indicate its nuclear localization. Combining this localization with the well-characterized functions of CBP family proteins, we hypothesize that *CaCBP2* may modulate pepper immune response pathways—thereby influencing plant resistance to *P. capsici*—by participating in transcriptional processes or mRNA processing. However, the specific functional mode and molecular mechanism of *CaCBP2* in pepper resistance to *P. capsici* remain completely uncharacterized.

Based on this hypothesis, we centered our study on *CaCBP2*: first, we characterized its expression dynamics in response to *P. capsici* infection; next, we dissected its functional role in pepper resistance to *P. capsici* using virus-induced gene silencing (VIGS) and heterologous overexpression; then, we elucidated its regulatory effects on the antioxidant system and defense hormone metabolism via physiological assays, while identifying its downstream target genes through transcriptome profiling; ultimately, we uncovered the molecular mechanism underlying *CaCBP2*-mediated pepper resistance to *P. capsici*. This study not only fills critical knowledge gaps in the functional characterization of the CBP family in Solanaceous crops but also provides a theoretical foundation for molecular breeding of *P. capsici*-resistant pepper varieties.

## 2. Results

### 2.1. Gene Structure, Subcellular Localization, and P. capsici-Induced Expression Characteristics of CaCBP2

**Figure 1:**
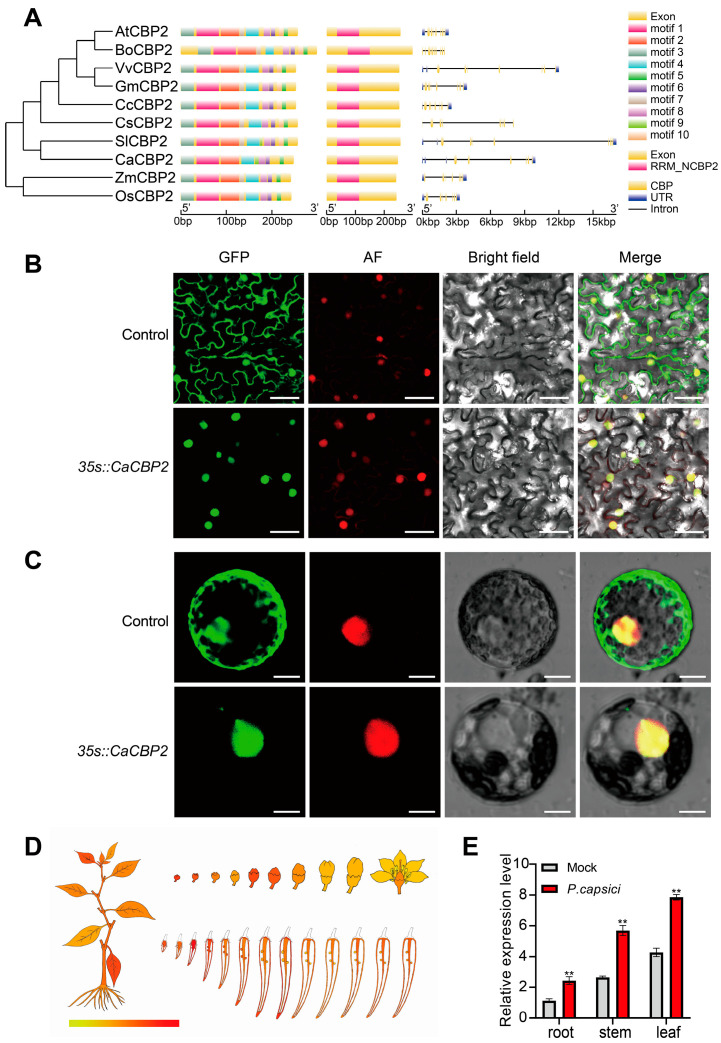
Gene structure, subcellular localization, and *P. capsici*-induced expression pattern of *CaCBP2*. (**A**) Phylogenetic tree, conserved motifs, domains, and gene structures of CBP genes from 10 species. (**B**) Subcellular localization of *CaCBP2*-eGFP in *Nicotiana benthamiana*(*N. benthamiana*) leaf epidermal cells (from left to right: eGFP signal, autofluorescence (AF), bright field, merged image of three channels), scale bar = 50 um. (**C**) Subcellular localization of *CaCBP2*-eGFP in *N. benthamiana* protoplasts (from left to right: eGFP signal, autofluorescence (AF), bright field, merged image of three channels), scale bar = 200 um. (**D**) Tissue expression pattern of *CaCBP2* in pepper. (**E**) Relative expression level changes in *CaCBP2* in roots, stems, and leaves after *P. capsici* infection. Data are presented as mean ± standard deviation (n = 3); Student’s *t*-test compared with the 0 h control, ** *p* < 0.01.

The pepper CBP gene family is characterized by strong functional specificity, a small number of members, and low homology within the family. In this study, phylogenetic analysis of CBP genes from pepper and 9 other species showed that pepper CBP genes have the highest homology with tomato (*Solanum lycopersicum*) CBP genes, followed by dicotyledonous plants such as citrus (*Citrus clementina*) and cucumber (*Cucumis sativus*), and the lowest homology with monocotyledonous plants such as maize (*Zea mays*) and rice (*Oryza sativa*). The length of CBP protein sequences from the 10 tested plants ranges from 244 to 299 amino acids, all containing 10 conserved motifs and an RNA recognition domain (RRM_NCBP2). Among them, motif 10 is unique to pepper and tomato, while the pepper CBP protein lacks motif 9. Based on the above sequence characteristics and evolutionary relationships, this pepper CBP gene was named *CaCBP2*. Further analysis showed that the length of CBP genes varies significantly among different species (1993–17,048 bp), all containing multiple introns, exons, and untranslated regions (UTRs), showing obvious species specificity (Figure 1A). The primer sequences used in this study are provided in Supplementary Table S1. The sequences of the motifs predicted from these DEGs are provided in Supplementary Table S2.

To verify the prediction by WoLF PSORT that *CaCBP2* is localized in the nucleus, a *CaCBP2*-EGFP fusion expression vector was constructed and transiently expressed in *N. benthamiana* leaves via Agrobacterium-mediated transformation. Protoplasts were prepared for laser confocal microscopy observation. Unlike the whole-cell diffuse signal of the empty EGFP vector, the green fluorescence of *CaCBP2*-EGFP was specifically enriched in the nucleus (Figure 1B,C), confirming that *CaCBP2* is a nuclear-localized protein.

The tissue expression pattern of *CaCBP2* was obtained from the pepper genome database PepperHub (Figure 1D), and the results showed that its expression level was the highest in leaves. qRT-PCR was used to detect the expression dynamics of *CaCBP2* in pepper roots, stems, and leaves after *P. capsici* infection. It was found that the transcriptional level of this gene was significantly higher than that of the uninoculated control after inoculation and reached a peak in leaves (Figure 1E), indicating that *CaCBP2* is significantly induced by *P. capsici* infection and may be involved in pepper immune responses.

### 2.2. Silencing of CaCBP2 Enhances Pepper Resistance to P. capsici 

**Figure 2:**
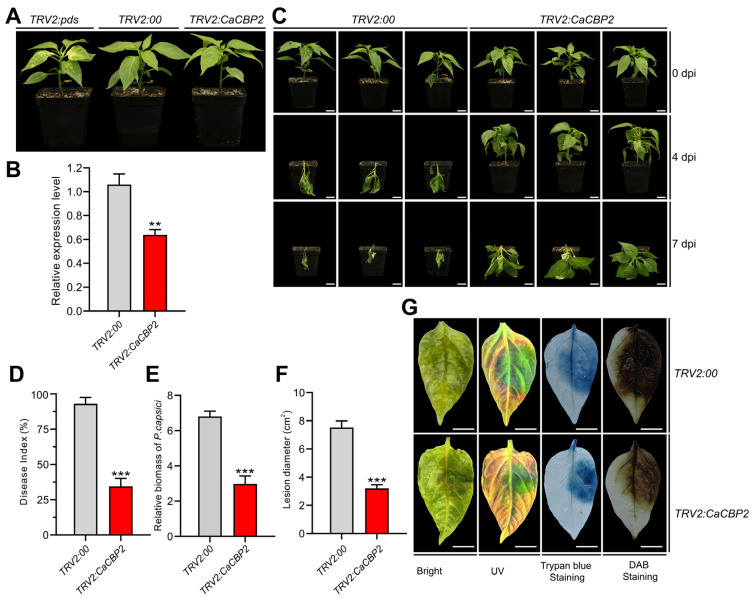
Silencing *CaCBP2* enhances pepper resistance to *P. capsici*. (**A**) Phenotypes of VIGS plants (*TRV2:00*: empty vector control; *TRV2:CaPDS*: photobleaching positive control; *TRV2:CaCBP2*: gene-silenced plants), scale bar = 1 cm. (**B**) Relative expression level of *CaCBP2* in silenced plants. (**C**) Phenotype comparison of plants at 0, 4, and 7 days after *P. capsici* inoculation, scale bar = 1 cm. (**D**) Disease index statistics 10 days after root irrigation inoculation. (**E**) Relative pathogen biomass (qPCR detection) 4 days after root irrigation inoculation. (**F**) Lesion area statistics 48 h after leaf inoculation. (**G**) H_2_O_2_ accumulation (DAB staining) and cell death (trypan blue staining) 48 h after leaf inoculation, scale bar = 1 cm. Data are presented as mean ± standard deviation (n = 3); Student’s *t*-test compared with the *TRV2*:00 control, ** *p* < 0.01, *** *p* < 0.001.

*CaCBP2* was demonstrated to be significantly upregulated upon *P. capsici* infection, implying its potential involvement in the immune response of pepper plants. In this study, *CaCBP2*-silenced pepper plants were generated using the Tobacco rattle virus (TRV2)-mediated virus-induced gene silencing (VIGS) approach. Four weeks after Agrobacterium tumefaciens infiltration, the positive control plants (*TRV2:CaPDS*) exhibited a typical photobleaching phenotype (Figure 2A), which verified the efficiency of the VIGS system. Quantitative real-time polymerase chain reaction (qRT-PCR) assays further confirmed that the transcriptional level of *CaCBP2* in the silenced plants was drastically reduced compared with the control group (Figure 2B).

Subsequently, both the silenced and control plants were inoculated with *P. capsici* via root drenching. At 4 and 7 days post-inoculation (dpi), the *CaCBP2*-silenced plants displayed remarkably attenuated disease symptoms relative to the *TRV2:00* empty vector control plants (Figure 2C). Specifically, the disease index of the silenced plants was significantly decreased (Figure 2D), accompanied by a marked reduction in pathogen biomass (Figure 2E). Consistent with these findings, the leaf inoculation assay showed that the lesion area on the leaves of *CaCBP2*-silenced plants was notably diminished, along with the alleviated accumulation of hydrogen peroxide and reduced extent of cell death (Figure 2F,G).

Collectively, these results indicate that *CaCBP2* positively regulates pepper resistance against *P. capsici*.

### 2.3. Transient Expression of CaCBP2 in N. benthamiana Enhances Susceptibility to P. capsici

**Figure 3:**
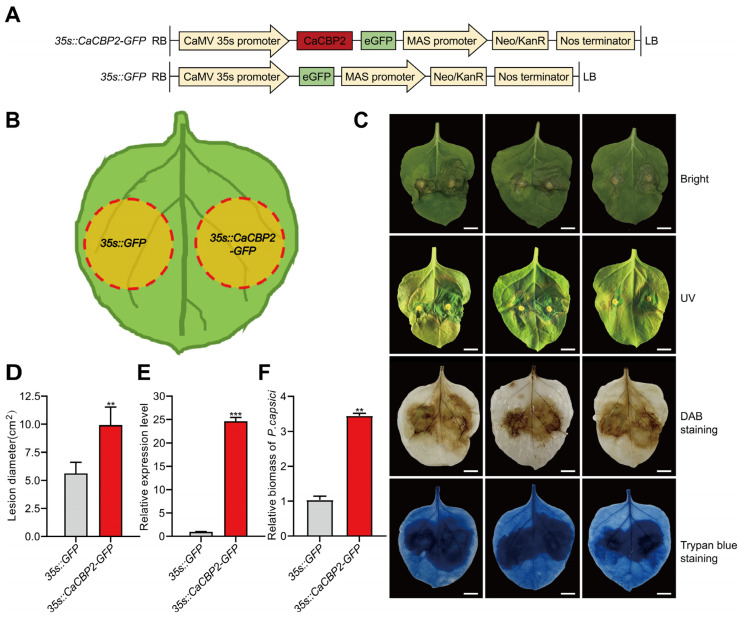
Transient expression of *CaCBP2* in *N. benthamiana* enhances susceptibility to *P. capsici*. (**A**) Schematic diagram of the *CaCBP2* expression vector driven by the CaMV35S promoter. (**B**) Design of transient transformation of tobacco leaves (left: empty vector; right: *CaCBP2* expression vector). (**C**) Lesion phenotypes and trypan blue staining (upper, dead cells) and DAB staining (lower, H_2_O_2_ accumulation) 48 h after *P. capsici* inoculation, scale bar = 1 cm. (**D**) Quantitative analysis of lesion area on the left and right sides of leaves. (**E**) Relative expression level of *CaCBP2* on the left and right sides of leaves. (**F**) Relative biomass of *P. capsici* on the left and right sides of leaves. Data are presented as mean ± standard deviation (n = 3); Student’s *t*-test compared with the empty vector control, ** *p* < 0.01, *** *p* < 0.001.

To rapidly identify the function of *CaCBP2*, we constructed a *CaCBP2* expression vector driven by the cauliflower mosaic virus 35S (CaMV35S) promoter (Figure 3A) and separately injected the empty vector and the *CaCBP2* expression vector into the left and right sides of *N. benthamiana* leaves, respectively (Figure 3B). Sixty hours after injection, the leaves were inoculated with *P. capsici*. Forty-eight hours post-inoculation, it was observed that the lesion area on the right side (*CaCBP2* expression) was significantly larger than that on the left side (empty vector control). Trypan blue and DAB staining results showed that the area of dead cells (dark blue) and hydrogen peroxide accumulation (brown) on the right side were significantly larger than those on the left side (Figure 3C). Statistical analysis of the lesion area further confirmed that the right side was significantly greater than the left (Figure 3D). qRT-PCR analysis revealed that the expression level of *CaCBP2* (Figure 3E) and the pathogen biomass (Figure 3F) on the right side were significantly higher than those on the left side. These results indicate that transient expression of *CaCBP2* in *N. benthamiana* enhances susceptibility to *P. capsici* infection.

### 2.4. Stable Overexpression of CaCBP2 in Tomato Enhances Susceptibility to P. capsici

**Figure 4:**
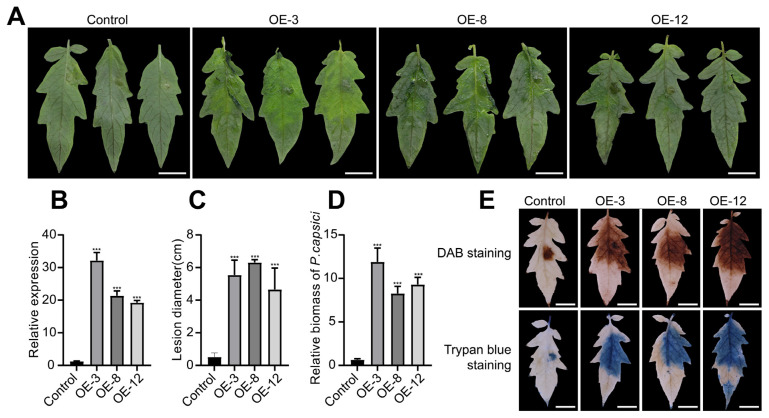
Stable overexpression of *CaCBP2* in tomato enhances susceptibility to *P. capsici*. (**A**) Leaf symptoms 48 h after *P. capsici* inoculation, scale bar = 1 cm. (**B**) Relative expression level of *CaCBP2* in wild-type (WT) and overexpression lines (qRT-PCR). (**C**) Lesion area statistics after inoculation. (**D**) Relative pathogen biomass statistics. (**E**) H_2_O_2_ accumulation (DAB staining, upper) and cell death (trypan blue staining, lower) after inoculation, scale bar = 1 cm. Data are presented as mean ± standard deviation (n = 3); Student’s *t*-test compared with WT, *** *p* < 0.001.

T3 generation homozygous lines of tomato stably overexpressing *CaCBP2* were obtained via Agrobacterium-mediated transformation. Three high-expression lines (Figure 4B) were selected for disease resistance evaluation. Seventy-two hours after inoculating detached leaves of wild-type and overexpression lines with *P. capsici*, the lesion area of overexpression plants was significantly increased (Figure 4A,C), the pathogen biomass was extremely significantly increased (Figure 4D), and H_2_O_2_ accumulation and cell death were weakened (Figure 4E). These results indicate that overexpression of *CaCBP2* in tomato enhances susceptibility to *P. capsici*.

### 2.5. Physiological Mechanism of CaCBP2-Mediated Pepper Resistance to P. capsici

To explore the physiological regulatory mechanism of *CaCBP2*, the following indices were determined: H_2_O_2_ content, DPPH free radical scavenging rate, O_2_^−^ free radical scavenging rate, POD activity, SOD activity, MDA content, and proline content. The change trends of each index were consistent before and after *P. capsici* inoculation, but the differences between silenced plants (*TRV2:CaCBP2*) and the control (*TRV2*:00) were more significant after inoculation.

**Figure 5:**
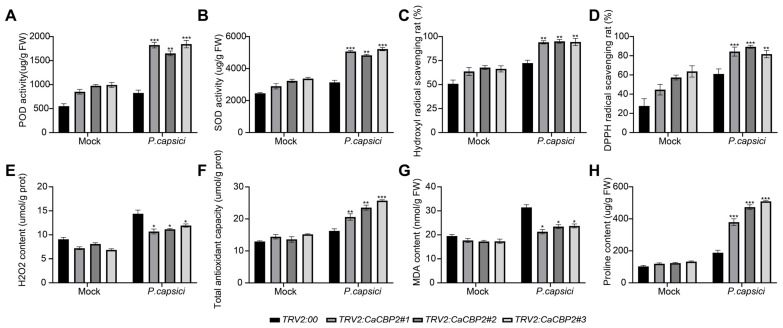
Effects of silencing *CaCBP2* on the antioxidant system and osmotic regulatory substances under *P. capsici* stress. (**A**) Difference in peroxidase (POD) activity. (**B**) Difference in superoxide dismutase (SOD) activity. (**C**) Difference in hydroxyl radical scavenging rate. (**D**) Difference in DPPH free radical scavenging rate. (**E**) Difference in hydrogen peroxide (H_2_O_2_) content. (**F**) Difference in total antioxidant capacity. (**G**) Difference in malondialdehyde (MDA) content. (**H**) Difference in proline content. Data are comparisons between the *TRV2*:00 control and *TRV2:CaCBP2*-silenced plants before and after pathogen inoculation. Data are presented as mean ± standard deviation (n = 3); Student’s *t*-test compared with the empty vector control, * *p* < 0.05, ** *p* < 0.01, *** *p* < 0.001.

Specifically, after inoculation, the POD activity (Figure 5A), SOD activity (Figure 5B), DPPH scavenging rate (Figure 5C), O_2_^−^ scavenging rate (Figure 5D), and proline content (Figure 5H) of *TRV2:CaCBP2* plants were significantly higher than those of the control; while the H_2_O_2_ content (Figure 5E) and MDA content (Figure 5G) were significantly lower than those of the control (Figure 5F shows the difference in total antioxidant capacity). These results indicate that silencing *CaCBP2* can regulate reactive oxygen species (ROS) metabolism and lipid peroxidation levels by improving antioxidant enzyme activity and enhancing free radical scavenging capacity, and *P. capsici* infection amplifies these differences.

### 2.6. Silencing CaCBP2 Increases the Content of Key Hormones Under P. capsici Stress

**Figure 6:**
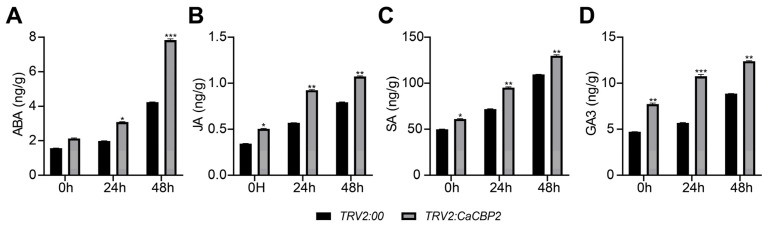
Silencing *CaCBP2* increases the content of key hormones under *P. capsici* stress. (**A**) Change in abscisic acid (ABA) content. (**B**) Change in jasmonic acid (JA) content. (**C**) Change in salicylic acid (SA) content. (**D**) Change in gibberellin (GA_3_) content. Data are comparisons between the *TRV2*:00 control and *TRV2:CaCBP2*-silenced plants at 0, 24, and 48 h after inoculation. Data are presented as mean ± standard deviation (n = 3); Student’s *t*-test compared with the empty vector control, * *p* < 0.05, ** *p* < 0.01, *** *p* < 0.001.

The contents of ABA, JA, SA, and GA_3_ in pepper at 0 h, 24 h, and 48 h after *P. capsici* inoculation were determined. Compared with the *TRV2:00* control, the contents of the four hormones in *TRV2:CaCBP2*-silenced plants were significantly increased at all time points, and the differences gradually expanded with the extension of inoculation time (Figure 6A–D). These results indicate that silencing *CaCBP2* can promote the accumulation of key hormones under *P. capsici* stress and regulate plant hormone metabolism to respond to infection.

### 2.7. Mining Hormone-Associated Hub Genes Mediated by CaCBP2 Based on WGCNA

**Figure 7:**
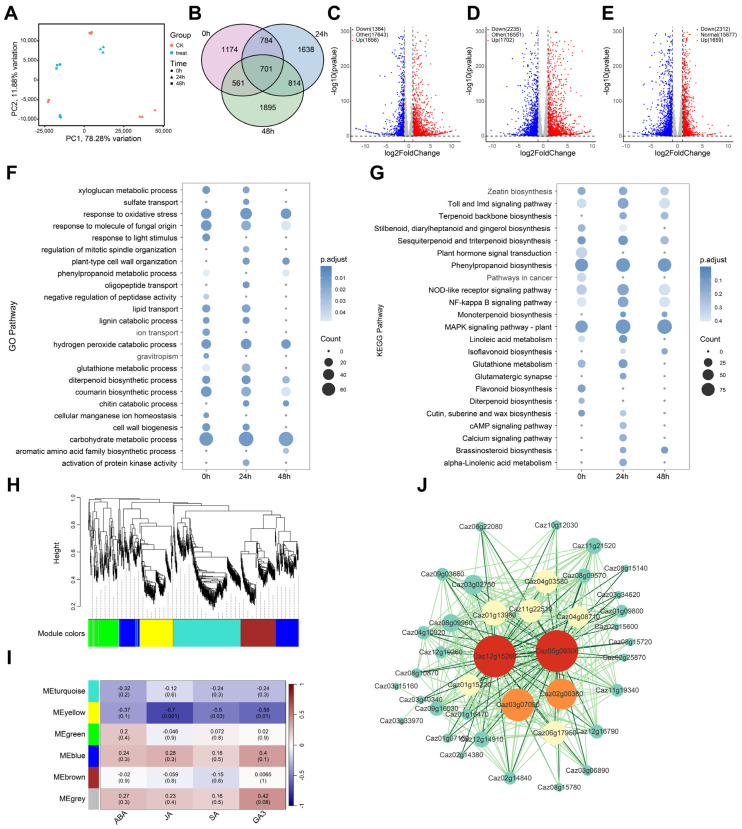
Transcriptome dynamics and hormone-associated gene networks of *CaCBP2*-mediated pepper blight resistance response. (**A**) PCA analysis of silenced CBP treatment and control after *P. capsici* inoculation at 0, 12, and 24 hpi. (**B**–**D**) Volcano plots of DEGs at the three time points, pink represents 0 h, blue represents 24 h, and green represents 48 h. (|log_2_FoldChange| > 1 and padj < 0.05). (**E**) Venn diagram of DEGs at the three time points. (**F**) GO enrichment analysis (circle size represents the number of genes in the pathway). (**G**) KEGG enrichment analysis (circle size represents the number of genes in the pathway). (**H**) WGCNA gene clustering and module division. (**I**) Correlation heatmap between gene co-expression networks and ABA, JA, SA, and GA_3_ contents. (**J**) Blue module gene network (weight > 0.95, circle size represents the number of gene interactions; red: hub genes; orange: secondary hub genes).

Transcriptome analysis was performed on samples with silenced *CaCBP2* at 0 h, 24 h, and 48 h after *P. capsici* inoculation. PCA showed that samples within groups were well clustered and separated between groups (Figure 7A). At the three time points, 1856 upregulated/1364 downregulated genes (0 h), 1702 upregulated/2235 downregulated genes (24 h), and 1659 upregulated/2312 downregulated genes (48 h) were identified, respectively, among which 701 genes were common DEGs (Figure 7B–E).

GO enrichment analysis showed that DEGs were enriched in biological processes such as “oxidative stress response”, “hydrogen peroxide catabolism”, and “response to fungal molecules”; genes related to “plant-type cell wall organization” and “chitin catabolism” were gradually expressed from 0 to 24 h and stably expressed from 24 to 48 h (Figure 7F). KEGG enrichment analysis showed that DEGs were enriched in pathways such as “zeatin biosynthesis”, “isoflavonoid biosynthesis”, and “MAPK signaling pathway”; genes related to “brassinosteroid biosynthesis” and “terpenoid backbone biosynthesis” were gradually expressed from 0 to 48 h (Figure 7G). The above pathways are involved in defense by strengthening the cell wall and synthesizing antibacterial substances.

WGCNA clustered the 701 DEGs into 5 modules, among which the blue module was positively correlated with hormone contents (Figure 7H,I). Supplementary Table S3 provides detailed information on the members and functional annotations of each WGCNA module. A gene network was constructed for the blue module, and *Caz12g15260* (C2 domain protein, involved in Ca^2+^ signaling and disease resistance defense) and *Caz05g09300* (Bet_v_1 domain protein, involved in secondary metabolism and stress response) were identified as core hub genes (Figure 7J).

## 3. Discussion

Research on the CBP gene family in plants is relatively scarce, with only sporadic reports on its involvement in biotic and abiotic stress responses in Arabidopsis thaliana and other species, while relevant studies in Solanaceous crops remain absent [37,38]. This study focused on *CaCBP2*, a cap-binding RNA-binding protein in pepper (*Capsicum annuum*). This protein is localized in the nucleus, and its expression is significantly induced by *P. capsici* infection. Functional validation was performed using virus-induced gene silencing (VIGS) and heterologous overexpression techniques. The results showed that silencing *CaCBP2* significantly enhanced pepper resistance to *P. capsici*, whereas heterologous overexpression of this gene in tomato increased plant susceptibility. These findings confirm that *CaCBP2* acts as a negative regulator of the pepper immune response against *P. capsici*. In addition, silencing *CaCBP2* enhanced the defensive physiological responses of pepper plants. Based on transcriptome data, we tentatively speculate that the silencing of *CaCBP2*—as a cap-binding RNA-binding protein—may trigger transcriptome reprogramming, thereby participating in the activation of key disease resistance pathways. However, this association requires further verification to clarify whether it is a direct regulation or indirect effect.

The nuclear localization of *CaCBP2* is highly consistent with the functional prediction that it may be involved in transcriptional or post-transcriptional regulation. As a homologous component of the nuclear cap-binding complex and a cap-binding RNA-binding protein, *CaCBP2* has a structural basis for participating in the regulation of mRNA processing, export, or stability. Thus, interpreting it as a potential transcriptional repressor or mRNA processing regulator is reasonable. Nevertheless, it should be emphasized that this mechanistic link is currently only a hypothesis, lacking support from direct experimental evidence such as chromatin immunoprecipitation (ChIP) and RNA immunoprecipitation (RIP) assays. This study found that the expression of *CaCBP2* is strongly induced by pathogen infection, suggesting that it is specifically recruited during host immune reprogramming. Notably, contrary to the general assumption that “most induced genes exert positive regulatory effects”, genetic evidence from this study confirms that *CaCBP2* actually functions as a negative regulator. This phenomenon is not uncommon in plant immune systems: some induced genes encode negative regulators to finely tune immune intensity and avoid growth costs caused by excessive immune activation. *CBP60a* in *Arabidopsis* has also been reported as a negative immune regulator [36], sharing certain functional similarities with *CaCBP2*. However, the association between their regulatory modes is only a preliminary speculation without direct evidence to support it.

Phenotypic analysis showed that *CaCBP2*-silenced plants exhibited significantly reduced lesion size, decreased pathogen biomass, and attenuated hydrogen peroxide (H_2_O_2_) accumulation and cell death at the infection site, while overexpressing plants displayed the completely opposite phenotypes. This cross-species gene function complementation experiment further supports the negative regulatory role of *CaCBP2* in pepper resistance to *P. capsici*. However, the molecular mechanisms underlying these phenotypes require more rigorous evaluation: the observed disease resistance phenotypes may either result from transcriptional regulatory changes directly mediated by *CaCBP2* or secondary effects of stress signals triggered by downstream signal transduction, which are difficult to distinguish completely. Therefore, future studies should focus on identifying the direct molecular targets of *CaCBP2* and clarifying the regulatory cascade to establish the direct association between phenotypes and regulatory mechanisms. Concurrent physiological data showed that silencing *CaCBP2* significantly enhanced antioxidant system activity, characterized by increased peroxidase (POD) and superoxide dismutase (SOD) activities, improved free radical scavenging capacity, promoted the accumulation of the osmotic regulatory substance proline, and reduced the contents of oxidative damage markers such as H_2_O_2_ and malondialdehyde (MDA). This indicates that *CaCBP2* may inhibit the activation of antioxidant mechanisms under normal conditions, thereby increasing plant sensitivity to pathogen-induced oxidative stress. This phenotype also needs to be verified in combination with direct targets to rule out the interference of secondary effects.

Hormone analysis results showed that silencing *CaCBP2* promoted the accumulation of abscisic acid (ABA), jasmonic acid (JA), salicylic acid (SA), and gibberellin (GA_3_) after *P. capsici* infection. However, currently, only differences in hormone contents can be confirmed, and the direct regulatory relationship between *CaCBP2* and these hormones cannot be clarified. To resolve this association, future studies should quantify the specific marker genes of each hormone pathway (e.g., PR1 for the SA pathway and PDF1.2 for the JA pathway) and combine them with target verification experiments to determine whether *CaCBP2* directly acts on the core components of these hormone pathways, thereby confirming their regulatory relationship. Transcriptome analysis results showed that 701 consistently differentially expressed genes were significantly enriched in defense-related pathways such as oxidative stress response and phenylpropanoid biosynthesis. The overall upregulation of these genes provides molecular-level support for the role of *CaCBP2* as an upstream inhibitor of immune signals, but the possibility that these gene expression changes are secondary regulatory effects cannot be ruled out.

Weighted gene co-expression network analysis (WGCNA) combined with hormone data successfully identified two key hub genes in the regulatory network: *Caz12g15260* (encoding a C2 domain-containing protein) and *Caz05g09300* (encoding a Bet_v_1 domain-containing protein). Among them, C2 domain-containing proteins are usually involved in calcium-dependent signal transduction and may link the function of *CaCBP2* to calcium signaling in plant immunity [39]; while Bet_v_1 domain-containing proteins are closely associated with stress responses and secondary metabolism [40,41]. Their core position in the co-expression network suggests that they may be downstream nodes in the *CaCBP2*-mediated regulatory pathway and participate in the formation of disease resistance phenotypes through synergistic regulation, but the specific regulatory relationship requires further verification in subsequent experiments.

In conclusion, this study clarifies that *CaCBP2* is a novel negative regulator of the pepper immune response against *P. capsici*, which ultimately weakens plant resistance to *P. capsici* by inhibiting antioxidant system activation, defense hormone accumulation, and defense gene expression. This finding fills the research gap in the disease resistance function of CBP proteins in important economic crops and provides important clues for the subsequent functional analysis of the identified hub genes.

## 4. Materials and Methods

### 4.1. Gene Structure and Conserved Sequence Analysis

Pepper (*Capsicum annuum*) genome data were obtained from the Pepper Genomics Database (http://ted.bti.cornell.edu/cgi-bin/pepper/index, accessed on 1 January 2026). Genome and protein sequences of tomato (*Solanum lycopersicum*), *Arabidopsis thaliana*, *Brassica oleracea*, *Vitis vinifera*, *Glycine max*, *Citrus clementina*, *Cucumis sativus*, *Zea mays*, and *Oryza sativa* were downloaded from EnsemblPlants (https://plants.ensembl.org/index.html, accessed on 1 January 2026). BLAST v2.16.0 (E-value < 1 × 10^−10^) was used to identify the most similar sequences to pepper CBP proteins in each species. Multiple sequence alignment was performed using Cluster function of MEGA 11 software (Tempe, AZ, USA) [42], and a phylogenetic tree was constructed by the Neighbor-Joining method with 1000 bootstrap replicates. Conserved domains were predicted using the NCBI Batch Web-CD-Search Tool (https://www.ncbi.nlm.nih.gov/Structure/bwrpsb/bwrpsb.cgi, accessed on 1 January 2026), and motif analysis was conducted based on the MEME suite (https://meme-suite.org/meme/tools/fimo, accessed on 1 January 2026). Visualization of gene structure was achieved using the Gene Structure Display Server (https://gsds.gao-lab.org/, accessed on 1 January 2026).

### 4.2. Plant Materials and Growth Conditions

The plant materials used in this study were pepper cultivar ‘Zhangshugang’ (*Capsicum annuum* ‘Zhangshugang’), tomato cultivar ‘Alisa Craig’ (*Solanum lycopersicum*), and *N. benthamiana*. All plants were grown in an artificial climate chamber with a photoperiod of 16 h light (24 °C)/8 h dark (22 °C), light intensity of 400 μmol·m^−2^·s^−1^, and relative humidity maintained at approximately 70%. Healthy plants with consistent growth status at the 6–8 leaf stage were selected for inoculation experiments.

### 4.3. Pathogen Culture and Inoculation

The tested pathogen *P. capsici* was isolated and identified by the College of Plant Protection, Hunan Agricultural University. The strain was activated on PDA medium at 28 °C for 7 days, then transferred to V8 liquid medium and cultured in the dark at 28 °C for 3–5 days. Mycelia were collected, washed three times with sterile water, and zoospores were released by light induction for 3 days, cold shock at 4 °C for 30 min, and induction at 28 °C. The zoospore suspension was adjusted to 1 × 10^5^ spores/mL, and 10 mL per plant was used for root irrigation or 20 μL per leaf for drop inoculation. After inoculation, plants were cultured in an artificial climate chamber at 28 °C with relative humidity > 90% [43].

### 4.4. Subcellular Localization

A pSuper1300-*CaCBP2*-eGFP fusion expression vector was constructed and transformed into Agrobacterium tumefaciens strain GV3101. The bacterial solution was cultured with shaking at 28 °C in YEP medium containing the corresponding antibiotics until the OD_600_ reached 0.8–1.0. Bacteria were collected by centrifugation, resuspended in infiltration buffer (10 mM MgCl_2_, 10 mM MES, pH 5.6) containing 200 μM acetosyringone, and the OD_600_ was adjusted to 0.5. The recombinant bacteria were mixed in equal proportions with Agrobacterium carrying the nuclear marker protein (mCherry-H2B) and p19 helper protein, and incubated at room temperature for 3–5 h. Leaves of 4–7 week-old *N. benthamiana* were selected, and the mixed bacterial solution was injected via pressure infiltration. After incubation in the dark at 22–25 °C for 12–24 h, the leaves were transferred to normal photoperiod for 36–48 h. eGFP fluorescence signals were observed using a Zeiss LSM510 laser (Carl Zeiss, Oberkochen, Germany) confocal microscope (excitation wavelength 488 nm, emission wavelength 500–530 nm), with the empty vector pSuper1300-eGFP as the negative control. The experiment was independently repeated three times [44].

### 4.5. Protoplast Preparation

Tobacco leaves after transient expression were cut into approximately 2 cm × 2 cm segments and placed in freshly prepared protoplast enzyme solution, followed by enzymatic hydrolysis in the dark at 23 °C and 50 rpm for 12–14 h. Protoplasts were gently shaken to release, filtered to remove tissue residues, purified by centrifugation, and sections were prepared for confocal microscope observation [45,46].

### 4.6. Virus-Induced Gene Silencing (VIGS)

p*TRV2:CaCBP2*, p*TRV2:*00 (empty vector control), and p*TRV2:CaPDS* (positive control) were co-transformed with the helper vector *pTRV1* into Agrobacterium tumefaciens strain GV3101, respectively. Monoclones were expanded and cultured until the OD_600_ ≈ 1.0. Bacteria were collected by centrifugation, resuspended in inoculation buffer (10 mM MgCl_2_, 10 mM MES, 200 μM acetosyringone, pH 5.6), and the OD_600_ was adjusted to 1.0. *pTRV1* and each *pTRV2* bacterial solution were mixed in equal volumes and incubated at room temperature for 3 h. Pepper seedlings with fully expanded cotyledons were selected, and the mixed bacterial solution was injected into the abaxial surface of cotyledons via pressure infiltration. Inoculated plants were cultured in the dark at 22 °C for 24 h, then transferred to 25 °C with a 16 h light/8 h dark photoperiod for 4 weeks. The photobleaching phenotype of p*TRV2:CaPDS* plants was used as the positive control. The silencing efficiency of *CaCBP2* was verified by qRT-PCR, and successfully silenced plants were selected for subsequent inoculation experiments. All treatments were set with three biological replicates [47].

### 4.7. Tomato Genetic Transformation

The constructed overexpression vector pGATE1300-35S-*CaCBP2*-NOS was transformed into Agrobacterium tumefaciens strain EHA105 by electroporation. After verification by colony PCR and sequencing, the strain was expanded and cultured until the OD_600_ reached 0.6–0.8. Cotyledons of sterile tomato ‘Alisa Craig’ seedlings were used as explants, infected in infection medium (MS + 100 μM acetosyringone) for 4 min, and then transferred to co-culture medium (MS + 2 mg/L 6-BA + 0.1 mg/L IAA + 100 μM AS) for dark culture for 2 days. Explants were transferred to selection medium (differentiation medium containing 10 mg/L hygromycin and 500 mg/L cefotaxime) to induce resistant buds, with subculture every 2 weeks. When buds grew to 1–2 cm, they were cut and transferred to rooting medium (1/2 MS + 0.1 mg/L IBA + 10 mg/L hygromycin) for root induction. Genomic DNA of regenerated plants was extracted, positive transgenic plants were identified by PCR, and homozygous T2 generation lines were obtained by continuous selfing [48].

### 4.8. Tissue Staining

Trypan blue staining: Inoculated leaves were decolorized in lactophenol solution in a boiling water bath, transferred to 0.4% trypan blue staining solution for 3–4 h, then decolorized with trichloroacetaldehyde overnight, and observed after mounting with glycerol [49] DAB staining: Leaves were immersed in 1 mg/mL DAB solution (pH 3.8) for 4–12 h in the dark, decolorized with 95% ethanol, and stored in glycerol-PBS (1:1) preservation solution for observation.

### 4.9. Determination of Physiological Indices

Kits provided by Beijing Solarbio Science & Technology Co., Ltd. (Beijing, China) were used to determine the activities of catalase (CAT), superoxide dismutase (SOD), and peroxidase (POD), as well as the contents of malondialdehyde (MDA), proline, and total antioxidant capacity, following the manufacturer’s instructions. Each treatment was set with three replicates.

### 4.10. Hormone Content Analysis

Leaf samples at 0 h, 24 h, and 48 h after inoculation were collected, quickly frozen in liquid nitrogen, and stored at −80 °C. The contents of abscisic acid (ABA), salicylic acid (SA), jasmonic acid (JA), and gibberellin (GA_3_) were determined by Nanjing Ruiyuan Co., Ltd. (Nanjing, China) using high-performance liquid chromatography–tandem mass spectrometry (HPLC-MS/MS).

### 4.11. Transcriptome Sequencing and Analysis

Total RNA was extracted using the Eastep^®^ Super Total RNA Extraction Kit (Promega, Madison, WI, USA), and the quality and integrity were detected by a K5800 ultra-micro spectrophotometer (Drawell, Chongqing, China). Using ≥1 μg RNA as the starting material, mRNA was enriched with Oligo(dT) magnetic beads to construct a PE150 sequencing library, which was sequenced on the DNBSEQ-T7 platform. Raw data were quality-controlled by Fastp v0.24.1 [50], aligned to the pepper reference genome by HISAT2 v2.2.1 [51], and gene quantification was performed using feature Counts v2.0.8 [52]. Differential expression analysis was conducted using DESeq2 v1.44.0 [53] (|log_2_FC| > 1, padj <0.05). Functional enrichment analysis was based on clusterProfiler v4.12.2 [54], weighted gene co-expression network analysis (WGCNA) was implemented using WGCNA v1.73, the threshold was set to 10 [55], and network visualization was completed using Cytoscape v3.10.0 [56].

## Data Availability

The transcriptomic data generated in this study have been deposited in the National Genomics Data Center (NGDC) of China under the accession number PRJCA055035 (available at https://ngdc.cncb.ac.cn/). Other supporting data related to this study are available from the corresponding authors upon reasonable request.

## Supplementary Materials

The following supporting information can be downloaded at: https://www.mdpi.com/article/10.3390/plants15030381/s1, Table S1: Primers used in the present study. Table S2: Motif sequences predicted in the present study. Table S3: Information on Members and Annotations of Each Module in WGCNA.
